# Experiences of distress and gaps in government safety net supports among parents of young children during the COVID-19 pandemic: a qualitative study

**DOI:** 10.1186/s12889-023-16037-4

**Published:** 2023-06-07

**Authors:** Alyssa C. Mooney, Kaitlyn E. Jackson, Rita Hamad, Lia C. H. Fernald, Mekhala Hoskote, Wendi Gosliner

**Affiliations:** 1 Philip R. Lee Institute for Health Policy Studies, University of California, San Francisco, 490 Illinois St, San Francisco, CA 94158 USA; 2 Division of Community Health Sciences, School of Public Health, University of California, Berkeley, 2121 Berkeley Way, Room 5302, Berkeley, CA 94720 USA; 3 University of California, Berkeley-University of California, San Francisco Joint Medical Program, 570 University Hall MC #7360, 2018 Oxford Street, Berkeley, CA 94720 USA; 4 Division of Agriculture and Natural Resources, Nutrition Policy Institute, University of California, 1111 Franklin Street, Oakland, CA 94607 USA

**Keywords:** COVID-19 pandemic, Safety net programs, Mental health, Parental burnout, Childcare

## Abstract

**Background:**

The COVID-19 pandemic prompted rapid federal, state, and local government policymaking to buffer families from the health and economic harms of the pandemic. However, there has been little attention to families’ perceptions of whether the pandemic safety net policy response was adequate, and what is needed to alleviate lasting effects on family well-being. This study examines the experiences and challenges of families with low incomes caring for young children during the pandemic.

**Methods:**

Semi-structured qualitative interviews conducted from August 2020 to January 2021 with 34 parents of young children in California were analyzed using thematic analysis.

**Results:**

We identified three key themes related to parents’ experiences during the pandemic: (1) positive experiences with government support programs, (2) challenging experiences with government support programs, and (3) distress resulting from insufficient support for childcare disruptions. Participants reported that program expansions helped alleviate food insecurity, and those attending community colleges reported accessing a range of supports through supportive counselors. However, many reported gaps in support for childcare and distance learning, pre-existing housing instability, and parenting stressors. With insufficient supports, additional childcare and education workloads resulted in stress and exhaustion, guilt about competing demands, and stagnation of longer-term goals for economic and educational advancement.

**Conclusions:**

Families of young children, already facing housing and economic insecurity prior to the pandemic, experienced parental burnout. To support family well-being, participants endorsed policies to remove housing barriers, and expand childcare options to mitigate job loss and competing demands on parents. Policy responses that either alleviate stressors or bolster supports have the potential to prevent distress catalyzed by future disasters or the more common destabilizing experiences of economic insecurity.

## Background

Daily life changed abruptly for most U.S. families while they sheltered-in-place and adapted to new public health measures at the start of the COVID-19 pandemic in March, 2020. Early state and federal efforts to mitigate COVID-19 transmission mandated or recommended business and school closures, and 90% of schools were closed by April [1]. Simultaneously, two thirds of childcare centers closed or reduced capacity to limit transmission, and one third remained closed a year later [2]. Many working parents were consequently forced to leave children unsupervised, work fewer hours, or quit their jobs or school to make up for the gap in care, which in turn increased the economic burden on families [2–6]. Moreover, exposure to school and childcare closures and the concomitant shift in care burden to parents was greater among lower-income families and racial/ethnic minorities, potentially widening inequalities in children’s learning opportunities, and delaying parents’ ability to return to work [1, 2, 7].

The combination of prolonged economic hardship, social isolation, and the added stress of managing childcare, home schooling, and jobs, contributed to the disproportionate harms to the health and well-being of families with school-aged children [8–14]. Among families in California, 70% experienced childcare disruptions, which were associated with depressive symptoms, lower self-rated health, and food insecurity [15].

The pandemic prompted rapid federal, state, and local government policymaking in 2020–2021, which primarily focused on unemployment and food insecurity [16]. Across the United States, the Special Supplemental Nutrition Program for Women, Infants, and Children (WIC) was expanded [17], the Supplemental Nutrition Assistance Program (SNAP, i.e., food stamps) monthly allotments increased several times [18], and a new food assistance program, Pandemic Electronic Benefits Transfer (P-EBT) was created to replace school meals students missed during closures. Policymakers bolstered joint federal-state unemployment insurance programs, and enacted eviction moratoriums to temporarily shield families from homelessness and housing instability [19]. Other national measures included three federal stimulus check payments, expansion of and advanced deposits of the 2021 Child Tax Credit, and maximized benefits of the Earned Income Tax Credit. Despite continued need and rising inflation, most federal and state safety net measures were temporary and expired in 2021.

In spite of the government commitment to buffering families from the effects of the pandemic, it remains unclear the extent to which pandemic alleviation measures helped families as they navigated school and childcare closures, particularly those already facing socioeconomic disadvantages. Lower-income families who accessed the expanded child tax credit reported fewer depressive and anxiety symptoms, suggesting that mitigating economic instability had health protective effects [20]. However, minimal government support was provided to address pervasive childcare disruptions [16], and this was consistent across the world. Of 195 countries surveyed, just nine implemented any childcare support initiatives in response to the pandemic [21].

A growing body of literature suggests that supports were inadequate to address the destabilization experienced most profoundly by families with children during the pandemic, as parents reported greater increases in food and housing insecurity [13, 22, 23], household chaos [24], child maltreatment [12, 25, 26], and worsening mental health in the U.S. [12, 15, 24, 27, 28] and internationally [8, 11, 29].

It is possible that adverse health effects depend on the types of disruptions and misalignment with available supports. In the U.S., states were given broad flexibility in how they implemented most federal pandemic support programs, including determining who is eligible and for how much. In addition, state capacity to fill gaps in federal support depended upon how the pandemic impacted state revenue [30]. While many state budgets were hard hit by the economic downturn, others like California, where this study was conducted, saw revenue growth from progressive income taxes on insulated high income residents [31]. Taken together, there was wide variation in state responses to mitigate pandemic harms, layering on pre-existing geographic differences in worker protections as well as healthcare and other policies [30, 32]. In some states, there was no moratorium on evictions or utilities shut-offs, expansions to Medicaid access, or paid sick and family leave, while others implemented a range of policies to protect struggling families. Maximum weekly unemployment benefits ranged from $200 to $800 across states [32].

California implemented one of the most robust pandemic policy responses in the nation [32], particularly with regard to workplace protections such as paid sick and family leave, expanded workers compensation, protections against forced return to work, and provision of childcare for essential workers. In spite of these policy responses, a majority of California caregivers reported disruptions to employment and childcare [15].

There has been little attention to families’ perceptions of whether the pandemic safety net policy response was adequate, and what is needed to alleviate lasting harms to family well-being. Understanding key gaps in government supports based on families’ perspectives and experiences, can inform a more holistic public health and policy response to meet the needs of U.S. families and children in the wake of the COVID-19 pandemic, as well as future public health or economic crises. Through qualitative interviews with low-income families with young children in California during the first year of the pandemic, the current study aimed to capture the most prominent challenges and critical gaps in government support that may have driven distress.

## Methods

Semi-structured qualitative interviews were conducted with parents of young children in California, as a component of the larger Assessing California Communities’ Experiences with Safety Net Supports (ACCESS) Study. Details on this study have been described previously [15, 33, 34], and are summarized below as they relate to the present analysis.

### Participants and recruitment

ACCESS study staff recruited a convenience sample of caregivers who met the following criteria: (1) lived in California at the time of the interview, (2) had at least one dependent under 9 years of age, and (3) were likely eligible for the Earned Income Tax Credit (EITC) based on self-reported income and other demographic characteristics. The ACCESS Study was initially designed to examine barriers to EITC take-up [33], although study goals were augmented to address safety net supports more comprehensively due to the COVID-19 pandemic. Potential participants were recruited in partnership with community-based organizations including safety net programs, social services agencies, tax preparation services, and other local organizations, such as community colleges who had large numbers of students on CalWORKs, which is California’s Temporary Assistance for Needy Families (TANF) program.

These partners recruited participants via email and text messages to their clients, newsletters, office bulletin boards, and social media pages. Participants were also asked to share study information with friends and family members. All recruitment materials included a link to a web-based eligibility screening questionnaire and consent form. People determined eligible for the study were contacted by study staff and scheduled for an interview.

Data were collected from August 2020 to April 2021. Interviews that occurred from August 2020 to January 2021 (*n* = 135) included a semi-structured qualitative component, described below. Interviews were conducted by telephone or video conferencing software in English or Spanish.

Qualitative interview questions were developed with feedback from a Community Advisory Board, and interviewers were trained by WG. Participants were asked what life had been like for them and their families since the start of the COVID-19 pandemic, their experiences with safety net programs since that time, and what programs or services would be most helpful. Purposive sampling to construct an analytic subsample for this analysis. Participants were stratified based on race and ethnicity, tax filing status, and receipt of the EITC, in order to ensure racial/ethnic and socioeconomic variation, as well as variation in access and utilization of government safety net programs.

### Data analysis

Thirty interviews initially were selected for transcription in Dedoose. Since the random number generator selected just one interview that was conducted in Spanish, an additional four interviews were then randomly selected from among the Spanish language interviews to increase language representation, as prior work had shown that language was associated with safety net participation in this sample [33], bringing the sample to 34.

We conducted a thematic analysis, which sought to construct themes as “central organizing concepts” [35]. First, the analysis team read all transcripts to become familiar with their content, and established an initial topic-based codebook, based on types of pandemic-related challenges and support programs discussed by participants. This was combined with inductive coding of all transcripts, to help identify patterns in the data that cut across topics, such as emotional experience. We conducted an iterative process of sorting codes into potential themes, reviewing and discussing collated extracts, and further coding to capture any additional data and subthemes within themes.

AM and WG individually coded and discussed transcripts in analysis meetings to reach consensus on the range of experiences and patterns being described within and across topics, to compare interpretations, resolve differences, interrogate assumptions that arose from differences in our backgrounds and experiences, and revise the codebook. Building upon initial discussions of patterns in domains, we began to identify themes related to the combination of pandemic challenges and gaps in support, emotions experienced, and the importance of the context of participants’ lives prior to the pandemic. Themes with collated data extracts were discussed in analysis meetings and with the broader study team for feedback.

The study team comprised California-based researchers with a range of expertise including social safety net programs, health and economic policy, school- and childcare-based interventions, social epidemiology, and family medicine. Some were parents of young children and some were not. By independently reading transcripts, attending to assumptions through writing and discussion, and regularly comparing and discussing findings, we sought to limit the influence of individual biases on results.

## Results

The 34 participants resided in 11 counties across California. Sociodemographic characteristics are shown in Table 1. Median income in the year prior to the pandemic was under $9,000, and the majority had some college education but no bachelor’s degree. Over half lived with three or more children, and about one third with three or more adults. Half were Latinx/Hispanic, and approximately one quarter were Black, and one quarter were white.

**Table 1:** Participant characteristics (*N* = 34)

Characteristics	Median (range) or N (%)
Age (years)	32 (21–45)
Income in 2019 (US$)	8,558 (0–36,783)
Self-identified female	31 (91.2)
Race/ethnicity	
Latinx/Hispanic	17 (50.0)
Non-Hispanic Black	8 (23.5)
Non-Hispanic White	9 (26.5)
Education	
High school or less	6 (17.7)
Some college/vocational/Associate’s	19 (55.9)
Bachelor’s or higher	9 (26.5)
Adults in home	
1	6 (17.7)
2	17 (50.0)
3+	11 (32.3)
Children in home	
1	9 (26.5)
2	6 (17.7)
3+	19 (55.8)

Sample was drawn from the larger Assessing California Communities’ Experiences with Safety Net Supports (ACCESS) Study, among those who were selected for semi-structured qualitative interviews

### Themes

We generated three main themes with nine sub-themes from participant interviews, presented in Table 2 and described in detail below. The first two themes involved positive and challenging experiences with government support programs, respectively. Participants reported that pandemic expansions helped to alleviate food insecurity, and recounted positive experiences with consolidated access to a range of supports through supportive counseling facilitated by community colleges. However, support for childcare, persistent pre-existing housing instability, and public support for parenting stressors were critical unmet needs. The third theme involved distress resulting from insufficient support for childcare and education disruptions that layered on pre-pandemic precarity.

**Table 2:** Themes generated from qualitative interviews

Theme	Quote
**Theme 1: Positive experiences with government support programs**	
***1.a Food support expansions***	*“We’re not actually worried about food, thank goodness, because the EBT is so helpful. It literally covers 100% of our food costs.”*
***1.b Consolidated supports through a responsive counselor***	*“…because that’s kind of all one package, we have like one person that we deal with that gets to know us.”*
**Theme 2: Challenging experiences with government support programs**	
***2.a Insufficient childcare and education supports***	*“…their education is kind of challenging and to keep up with this it’s either to spend a lot of money [on] a private teacher or to spend all your time just trying to teach the kids. If they, if there is any kind of extra help with this part too it would save me a lot of time to either work or study.”*
***2.b Persistent pre-pandemic housing instability***	*“I’m paying my rent late every month…I have missed work for medical conditions unrelated to COVID, so my reduction in hours that put us slightly behind is not caused by COVID, so they won’t help me. And I can’t seem to find anything else that will.”*
***2.c Lack of public support for parenting stressors***	*“I’m just a parent who’s sometimes stressed and sometimes might need a counseling session…to say like, ‘how do I manage? Like walk me through this, or my kids are really acting out right now because of the enclosure.’”*
**Theme 3: Distress resulting from insufficient support for disruptions**	
***3.a Precarious balance pre-pandemic***	*“…we had all these little supports that were all pulled together to make everything work and that was already a challenge. You know, we were stretched thin just keeping everything going.”*
***3.b Stress, overwhelm, and exhaustion***	*“…basically, it kind of became a situation of not sleeping. Where I would be a mom during the day and then not sleep at night to get all my work done and be a student…It’s been really, really stressful.”*
***3.c Guilt***	*“I think the thing that has impacted us most is our kids…we can only devote so much time to structured learning. And they’re suffering for it, greatly.”*
***3.d Sacrifices and stagnation***	*“I had to stop going to school myself…crushing to my soul.”*

### Positive experiences with government support programs

#### Food support expansions

With regard to pandemic program expansions, the vast majority expressed that food support, like increases to SNAP benefits and the availability of free school meals for pick up, alleviated the increase in food-related expenses with children home during the day. There was broad consensus that food program expansions were a source of support during the pandemic, although some mentioned the health risks of standing in food pantry lines or going to grocery stores, and challenges with coordinating meal pick-ups.

*“We’re not actually worried about food, thank goodness, because the EBT [SNAP] is so helpful. It literally covers 100% of our food costs…In 2019 we didn’t have, we never had enough money for food…My mom would go and stand in line at various food banks multiple times a week, just to cover the difference, but since the pandemic and now that our food stamps have gone up, we don’t actually need to utilize that.” (age 25–34, White)*

#### Consolidated supports through responsive counselors

Many participants recounted positive experiences with obtaining supports through their schools, particularly because community colleges offered one-stop-shops to access a range of services and aid from CalWORKs and other programs, through one responsive counselor in an otherwise fractionated safety net system. A few noted that the combination of supports that filled holes in the safety net, and noted that a supportive counselor enabled them to continue their degree programs despite the strains of the pandemic.

*“… the whole CalWORKs program that, you know, supports our school needs, and also helps with cash aid, and also helps with the CalFresh [SNAP], the food stamps. So, because that’s kind of all one package, we have like one person that we deal with that gets to know us. We know who to call, if we have questions or issues that need to be dealt with…” (age 25–34, White)*

*“CalFresh and CalWORKs also helped me stay motivated to stay in school…sometimes I don’t even have the time…But the counselors there too, like they help you out…if it wasn’t for my, you know, counselors through them, I probably would have been like, oh my god, I give up.” (age 25–34, Latinx/Hispanic)*

### Challenging experiences with government support programs

#### Insufficient childcare and education supports

However, many participants expressed frustrations with the lack of housing supports and childcare options to enable parents to continue to earn income and pursue degree programs. Almost all participants expressed a need for additional childcare support. This included supervision for young children while parents worked, and/or tutoring to prevent school-aged children from falling behind, given participants’ insufficient time, money, skills, or quiet space to support their children’s online coursework.

*“I mean, if they had a program for a tutor. That would help. Or if there was a program where they could go to daycare, just to do their homework, like from the hours of live instruction and then when that’s over, then come get them. Because, only because mine have, I don’t know, I would say a learning disability or behavioral. And they’re all together. Yeah. So it’s just a struggle, trying to find a quiet place.” (age 25–34, Black)*

*“…their education is kind of challenging and to keep up with this it’s either to spend a lot of money [on] a private teacher or to spend all your time just trying to teach the kids. If they, if there is any kind of extra help with this part too it would save me a lot of time to either work or study… that would help, I would make sure the kids are on the right track, not less than they’re, than the level they’re supposed to be, because of this situation.” (age 35+, White)*

Just two participants accessed pandemic programs for childcare. One received assistance through the essential workers program, which was helpful but short-lived:

*“While they are currently at a daycare, the funding that I have for the essential worker program is supposed to end at the end of this month…” (age 25–34, White*)

Another mentioned hearing about this program, but that it was implemented after she had already left her job to provide childcare.

Given the opportunity costs of formal employment, a few also suggested reimbursements for those who stay home to provide childcare and distance learning support. One participant received a county subsidy that provided funding for family and friends to provide in-home care, but which was an insufficient amount:

*“I’m in the process of getting my sister-in-law to help me watch my daughter. I wish they gave maybe bigger grants for these times to pay family members or someone else to watch your kids because right now they’re just going to pay her $3 an hour to watch her… that’s nothing.” (age 25–34, Latinx/Hispanic)*

#### Persistent pre-pandemic housing instability

The high cost of housing and lack of housing support services had been a major challenge for families long before the pandemic, and many lived in tight spaces or doubled up with extended family. Participants who had applied for rental support or affordable housing either prior to or during the pandemic expressed frustration that they were often placed on long waitlists and never heard back.

*“Housing is the hardest thing for us here in California.” (age 35+, Latinx/Hispanic)*

One participant stated that a pandemic housing grant through her city prevented her family from experiencing homelessness, yet in some cases eligibility specific to COVID-19 was at odds with the reality of housing instability that pre-dated the pandemic:

*“… for rental assistance, I’m told I need an eviction notice, but nobody’s passing out eviction notices right now, so I’m paying my rent late every month. And the only other program that I can find will only help if we’re struggling financially because of COVID. So I have missed work for medical conditions unrelated to COVID, so my reduction in hours that put us slightly behind is not caused by COVID, so they won’t help me. And I can’t seem to find anything else that will, so we’re just kind of in survival mode right now just trying to pay what we can, when we can.” (age 25–34, White)*

After losing her housing, she confronted a second barrier: landlords required that prospective tenants show evidence of income that equals or exceeds three times the monthly rent, and income support programs were not considered income for lease agreement purposes. Several participants mentioned a need for policy change addressing this hurdle to improve access to housing:

*“Honestly, I feel like there really needs to be a service that can help you waive the two and a half or three and a half times monthly rent thing for California because that’s what’s causing me to be homeless…I’m being forced out of county two hours, two plus hours away just because I can’t afford anything here technically, but I can.” (age 18–24, White)*

#### Lack of public support for parenting stressors

Given such acutely stressful and emotionally taxing experiences, some expressed that ongoing counseling services for parenting challenges is a critical gap in social safety net programs, which focus on material support and emergency situations. Several expressed that they wanted “someone to call” when they were stretched too thin, and needed emotional support and coping strategies for chronic stressors:

*“I can survive and get through it with even though it’s like a super struggle, super narrow. Like with the financial support… you can scrape by. But I think the biggest thing is mental health support, because you’re trying to do so much in such a difficult situation…if there were some sort of counselor I could talk to or someone who, you know, could help me figure out how to address like feelings of panic or anxiety…if CalWORKs or one of those programs was able to say we have someone for you guys to call and talk to when you’re having those moments. When you just want to break down and tear your hair out and cry in the corner…” (age 35+, Latinx/Hispanic)*

*“I don’t consider myself as having any like mental health care problems, but I’m just a parent who’s sometimes stressed and sometimes might need a counseling session or a referral to help me cope with like some parenting strategies… to say like, ‘how do I manage? Like walk me through this, or my kids are really acting out right now because of the enclosure.’ … which I think could be very helpful to me, and to many, many families who just need to talk to somebody. And I know that there are emergency services, … but it seems like if somebody’s calling like an emergency service hotline and it’s like a lot of that is a little too late, you know.” (age 35+, Black)*

### Distress resulting from insufficient support for disruptions

Parents were functioning with inadequate support and substantially increased burdens due to school and childcare facility closures. As a result, they reported high levels of stress, overwhelm, exhaustion, and guilt as they struggled to help their children through distance learning and social isolation, while their own work and educational requirements competed for their attention. Ultimately many quit their jobs or college degree programs to provide childcare and educational support.

#### Precarious balance pre-pandemic

Participants described their pre-pandemic lives managing finances and schedules that were very tightly balanced, with little room to maneuver. Many were enrolled in college degree programs in addition to working and raising young children. They described relying on carefully constructed systems and social supports to manage limited time and money, and to separate physical space in order to meet competing demands.

*“…even before the pandemic my husband and I were trying to manage school and raising our family and working part-time to support everything because student aid is not enough to raise a family all by itself. And, and so we had all these little supports that were all pulled together to make everything work and that was already a challenge. You know, we were stretched thin just keeping everything going.” (age 25–34, White)*

*“… before everything shut down, my two older children, my school-aged children were in the afterschool program at their school, so they were actually in school from 7:30 until six o’clock. This made it a lot easier, especially because my only other caregiver was my mom…my mom picked him up because I wasn’t going to get home in time…Afterschool helped them with their homework. My two younger babies were down the street from me at a daycare. When everything shut down, all of that shut down.” (25–34, White)*

#### Stress, overwhelm, and exhaustion

Parents described the additional workload they absorbed in caring for and educating their children when schools moved online and daycares closed or felt unsafe for health reasons. Young children required full-time supervision and older children needed help with distance learning, leaving parents limited time during the day for paid employment and training programs. A few sought night shifts; those who were able to work from home reported feeling mental and physical exhaustion from working late into the night after a full day of providing childcare:

*“… basically, it kind of became a situation of not sleeping. Where I would be a mom during the day and then not sleep at night to get all my work done and be a student… That has its own health consequences. Both mentally and physically. It’s been really, really stressful.” (age 35+, Latinx/Hispanic)*

Though many participants noted how daycare closures added to competing demands at home, others also discussed safety concerns with centers that remained open. They described weighing the risk of acquiring COVID-19 and its fallout, against the additional childcare workload if they dis-enrolled from daycare.

*“… I know that there are [childcare] providers open, but my level of comfort sending him somewhere has decreased dramatically. … I rather struggle with, you know, making sure he attends his school sessions and then if I need to like work late into the night. I’ll do that over one of us being exposed to COVID and then potentially like being in the hospital for an extended period of time.” (age 25–34, Black)*

Some continued jobs as essential workers and described the stress of scrambling to fill childcare gaps within their strained support systems. Participants reported that their children struggled immensely with distance learning and isolation, and often required a great deal of support to keep up with online courses and to manage the loss of structure and social interaction. Many also shared that their children had learning disabilities or special needs, and they had depended upon routines and specialized education support services provided by schools. They frequently reported feeling overwhelmed with trying to fill these additional roles, particularly in the context of meeting competing demands for their jobs and college coursework:

*“… suddenly having my school-aged children home and having to juggle my full-time job, that was just so overwhelming and having them home, I mean, I felt like I was in survival mode and I was just extremely overwhelmed. And then, particularly my oldest, he needs something much–a lot more structured than he was having with the virtual learning…since I work full-time and my partner doesn’t partake in very much of the childcare at all, and my mom being disabled, she couldn’t manage my two older children and my two younger babies … I just went into panic mode. Like what am I supposed to do with my children?” (age 25–34, White)*

Much of the increase in stress parents described was related to the convergence of competing demands into one physical space—their homes—when daycares, schools, and workplaces closed.

*“I’m working from home and I’m assisting him and so like I feel like my 8-hour really extends into like a 12-hour day just because I’m bouncing back and forth between assisting him, meeting with my clients, and then doing my paperwork later in the day. Whereas before, when I was going to work, I was able to do paperwork and meet with clients. And then once I got home, I was able to devote my time and attention to him.” (age 25–34, Black)*

This was particularly difficult for the many participants who lived in small apartments or shared housing with extended family because of high housing costs. Participants reported struggling to find quiet spaces where each member of the family could work without distraction, and the conflict arising from differences in family members’ needs, priorities, and patience related to home education:

*“So it’s been stressful having to do, do school and care for him and then on the side have my siblings also here like they need help with work —with school too— and they’re also pretty loud.” (age 18–24, Latinx/Hispanic)*

Pressures were heightened among participants who were pushed to live in more affordable areas far from work, which further complicated childcare and time constraints by extending commute times:

*“… in March, I got sick and we had to move, and I moved away from my child’s school, like a whole city like, like 30 minutes away from her school, so now it’s a little bit more difficult.” (age 25–34, Latinx/Hispanic)*

#### Guilt

Parents described developing new structures and systems to keep everyone afloat and prevent their children from falling behind in school. However, feelings of guilt were commonly reported among participants who observed their children struggling with distance learning and social isolation, while their own work and school demands competed for their attention. Some reported also feeling ill-equipped to provide the help their children needed with their courses. They reported worrying about protecting their families from health risks, as well as the effects of shutdowns on their children’s emotional well-being and social and educational development.

*“The jungle struggle is trying to manage the kids at home and then also trying to work and also get resources and go out and get groceries and, you know, there’s times where I felt guilty because I’ve had to take them with me to the grocery store and one of my daughter still sucks her fingers so it’s still like, oh my god, please don’t touch, don’t touch. And then I think just the guilt, I think, as a parent, trying to overcome that with them trying to help them navigate their little world of understanding why they can’t go out, why they can’t see their friends, why they can’t go see their cousin and do certain things.” (age 35+, Black)*

Many described how they were pulled in multiple directions by employers, college courses, and children, and felt as though meeting the needs of one was always to the detriment of another.

*“And then there is that pressure and also guilt of being like, here is this tiny human that just wants love and attention and affection from you and you’re trying to perform as a student and there is the pull of both directions. It’s like you feel like you [can] either be good at one or good at the other, but not at both, because there’s no clear delineation when you’re working from home, which job you’re working in.” (age 35+, Latinx/Hispanic)*

*“I think the thing that has impacted us most is our kids … They went from being in a highly structured school environment where they were, you know, engaged and learning, to being at home full-time and they’re bored and they’re annoyed, and they miss their friends and, you know, unfortunately because I’m a full-time employee and a part-time student and my partner is a full-time student, you know, we can only devote so much time to structured learning. And they’re suffering for it, greatly.” (age 25–34, White)*

One suggested that the emotional strain of feeling unable to support and protect their children as much as they would like to, while balancing the additional stressors and workloads that pandemic conditions imposed, was at the core of mental health struggles among mothers.

*“I feel like mental health for women, mothers, is really difficult right now, because as for me, like I want to be the best mother I could. But right now, it’s just very difficult to give them all the attention.” (age 25–34, Latinx/Hispanic)*

#### Sacrifices and stagnation

Many participants reported that they ultimately needed to quit their jobs or college degree programs to provide childcare. Some described the strains of having to put their goals on hold and halt progress towards their educational advancement and their family’s economic security while they worked to fill gaps in childcare, keep their families safe and healthy, and make sure their children did not fall behind in their classes:

*“… I need to be sitting literally next to my son to make sure that he’s following the, you know, protocol in the classroom…which also obstructs me from getting a job, so that way I can pay my bills and be able to move on financially.” (age 25–34, Latinx/Hispanic)*

*“I’d have to have them have a schedule, you know, for classes and make sure they’re, you know, doing what they need to do. You know, I had to stop going to school myself… a crushing to my soul …” (age 25–34, Black)*

## Discussion

This qualitative study of parents with lower-incomes caring for young children in California in 2020 and 2021 found that families were already stretched thin before the COVID-19 pandemic, managing a precarious balance of limited time, money, and space. Participants reported that while food security programs addressed increased expenses with children at home, supports for housing, childcare, distance learning, and the resulting distress were insufficient and failed to mitigate the harms of disruptions. This resulted in immense stress, exhaustion, and overwhelm; parental guilt about competing demands; and stagnation of their economic and educational advancement. Taken together, the emotional experiences and inadequate supports parents reported were reflective of parental burnout.

Participants’ stories aligned with studies showing that COVID-19 disruptions introduced additional parenting stressors such as increased childcare workloads, while simultaneously reducing resources such as social supports, resulting in an increase in parenting stress, exhaustion, and poor mental health compared to non-parents [8–11, 13, 14, 25]. Descriptions of distress were reflective of the emerging construct of parental burnout, which results from a chronic imbalance of parental demands with available resources [36, 37]. When parenting stressors chronically overwhelm available coping resources, it produces exhaustion in one’s parenting role; the feeling of not being as good a parent as before; resulting distress, shame, and guilt; feeling fed up; and ultimately emotional distancing from one’s children [37].

Though research on parental burnout is nascent, extant studies suggest it can result in a range of harmful outcomes for the parents experiencing burnout, and negatively impact relationships with partners and the health and well-being of their children [38–41]. In comparison to job burnout or depression, parental burnout is associated with more frequent suicidal ideation [39], and psychological forms of escape such as alcohol use [38]. Suicidal ideation and alcohol use both increased among parents during the pandemic [11].

Almost no research has been conducted to identify effective policies or interventions to prevent or address parental burnout [42]. Policy responses that either alleviate stressors or bolster supports as outlined by participants in this study, may have the potential to prevent parental burnout catalyzed by future disasters or the more common destabilizing experiences of acute or chronic economic insecurity [20]. Yet compared to the numerous and diverse set of pandemic response programs aimed at addressing economic precarity, there was limited support directly addressing the housing insecurity and loss of childcare mentioned by participants, and none to address emotional distress among parents. In California, programs and policies modified or created in response to the COVID-19 pandemic included at least 20 safety net policies addressing unemployment and food insecurity, but just 4 related to housing support and 4 addressing childcare and school closures (only 2 of which provided support for childcare) [16].

One state-level program funded childcare for up to 20,000 essential workers, but only from April to September 2020 [16]. Participants indicated that the essential workers program started too late and ended too early, that reimbursements were insufficient, and that safety concerns with daycare centers remained. In the ACCESS Study, from which the present analysis arose, 70% of parents reported childcare disruptions, which were associated with depression, poor self-rated health, and food insecurity [15].

However, following pandemic-related reductions in childcare facilities, promising structural reforms have begun to emerge with an increase in federal investment. In 2021, the American Rescue Plan Act provided unprecedented levels of funding support for childcare provider relief and recovery through childcare stabilization grants, and supplemental investments in the Child Care and Development Block Grant for families with young children. Given the flexibility to determine how funding will be used and disbursed, states have developed initiatives to reduce inequities in families’ access to care and eligibility for support, and to improve provider compensation, benefits, and payment rates [43].

With regards to gaps in housing supports, participant accounts reflected a misalignment between what few housing protections arose during the pandemic, and the dire lack of affordable housing that pre-dated it. The physical convergence of roles within crowded living spaces was a significant source of stress, and the need to live in more affordable peripheral areas exacerbated time constraints, with cascading effects. Since housing costs had created poor living conditions before COVID-19, pandemic responses like eviction moratoriums or COVID-19-specific stipulations for rental support were inadequate without increasing access to affordable housing. These findings may help to explain the lower reported frequency of pandemic housing disruptions, compared to childcare and employment, in the parent survey study [15]. Participants were facing housing insecurity and inadequate government supports before the pandemic, so were already doubling up with families and frequently relocating to stay housed. In the larger ACCESS Study, 8% of parents in the parent survey reported housing disruptions, associated with a higher likelihood of experiencing depression, poor self-rated health, and food insecurity [15].

Indeed, housing is now widely recognized as an important social determinant of mental and physical health, and health systems have begun to address housing instability as a health intervention, including Medicaid waivers for housing services in some states [44, 45]. In addition to a clear need for more affordable housing options, policy responses could address the barriers to entry reported by participants, including the requirement to show income equal to three times the monthly rent. At the time of writing, California Assembly Bill 12 was recently introduced to the state legislature to prohibit landlords from demanding security deposits for lease agreements which exceed two months’ rent. More research is needed to evaluate the mental health effects of these types of policy responses to improve housing security.

In addition to material supports, participants reported a desire for counselors to provide guidance and emotional support for parenting and child behavioral challenges that contributed to chronic stress, and to assist with navigating a fragmented safety net system on an ongoing basis. A few described successful models of receiving this form of support at their community colleges, where they accessed a range of government support programs. Participants’ desire to have “someone to call” when parenting pressures mounted aligns with an intervention shown to be effective to reduce burnout through active listening, participant sharing, and encouragement of participants’ feelings of worth and ability [42]. More broadly, case workers could incorporate screenings for parental burnout using validated measures [37], and provide referrals for mental and behavioral health resources, in addition to economic supports. Many participants reported that their children had special needs or behavioral challenges that were exacerbated by the pandemic, and are associated with greater risk of parental burnout [46]. Therefore, expanding school-based emotional and behavioral interventions that involve parents as well as their children may be an important policy area to target burnout and improve family well-being.

### Limitations

The findings of this qualitative study do not aim to be representative, but rather reflect the experiences and perspectives of a sample of parents with low incomes caring for young children in California. However, these accounts can help to inform policymaking and future larger-scale quantitative studies. The latter may build upon these findings by investigating trajectories of parental burnout and its effects using quantitative measures [37], as well as evaluating the effects of policies and programs to improve parents’ mental health.

## Conclusion

This sample of California parents of young children expressed gratitude for the food security programs that were expanded as a result of the pandemic. The study participants also expressed a need for improving government supports for childcare and the lack of affordable housing that pre-dated the pandemic, as well counseling services to address chronic stress and resulting parental burnout. Addressing these structural barriers is critical for protecting families’ mental health and well-being during future crises as well as non-crisis periods.

## Data Availability

The data generated and analyzed during the current study are not publicly available due to the sensitivity of information collected by participants, but are available from the corresponding author on reasonable request.
